# Ethnic Differences in Body Mass Index Trajectories from Adolescence to Adulthood: A Focus on Hispanic and Asian Subgroups in the United States

**DOI:** 10.1371/journal.pone.0072983

**Published:** 2013-09-05

**Authors:** Sandra S. Albrecht, Penny Gordon-Larsen

**Affiliations:** 1 Carolina Population Center, University of North Carolina, Chapel Hill, North Carolina, United States of America; 2 Department of Nutrition, Gillings School of Global Public Health, University of North Carolina, Chapel Hill, North Carolina, United States of America; NIDDK/NIH, United States of America

## Abstract

**Background:**

Compared to whites, U.S. Hispanics have higher obesity rates; U.S. Asians have lower rates. However Hispanics and Asians are each comprised of several ethnic subgroups that differ with respect to country of origin, immigration history, and geographic distribution across the U.S. Among adolescents, ethnic differences in obesity have been previously reported, but no studies have examined longitudinal change in body mass index (BMI) by Hispanic and Asian subgroup category to understand when and why these disparities emerge, especially during the critical transition between adolescence and adulthood.

**Methods:**

Using nationally-representative, longitudinal data from 1355 Hispanics (Mexican, Puerto Rican, Cuban, Central/South American, Other Hispanic), 520 Asians (Chinese, Filipino, Other Asian), and 5061whites from the National Longitudinal Study of Adolescent Health (Waves II–IV: 1996–2009), we used linear mixed spline models to examine whether Hispanic and Asian adolescent subgroups shared the same BMI trajectories as whites as they aged into adulthood. We also investigated the role of social and behavioral factors in explaining race/ethnic differences.

**Results:**

Among Hispanics, Mexican and Puerto Rican-origin individuals exhibited faster increases in BMI both in adolescence and in adulthood and these patterns were not attributable to the measured social and behavioral factors. There was also evidence of emerging disparities in Cuban males, and in Central/South Americans relative to whites. In contrast, Chinese, Filipino, and Other Asian adolescents had significantly lower BMI and slower BMI increases in adulthood compared to whites. In models adjusted for social and behavioral factors, Chinese-white and Other Asian-white differentials remained unexplained.

**Conclusions:**

Aggregate estimates of Hispanics and Asians mask important heterogeneity in BMI. A better understanding of weight dynamics early in the life course can inform how and when disparities emerge to better target prevention efforts.

## Introduction

Although obesity has impacted all segments of society, rates are high in Hispanic and low in Asian as compared to white adolescents and adults in the United States [1]–[3]. Disparities, especially in Hispanics, also appear to be exacerbating over time. For example, between 1986–1998, overweight prevalence in Hispanic adolescents increased 120% compared to 50% for whites [4].

Much of this literature examines Hispanics and Asians as single pan-ethnic groups despite the fact that each are comprised of several ethnicities that are heterogeneous with respect to country of origin, genetic ancestry, immigration history, and geographic distribution which likely contribute to variation in weight patterning. While there has been increasing recognition of Hispanic and Asian subgroup heterogeneity in health research [5], [6], data limitations have precluded their disaggregation. Nevertheless disentangling these pan-ethnic categories is necessary to better identify sub-populations at greatest risk.

Limited research on weight patterning by ethnic subgroup has been cross-sectional. In adults, Mexicans and Puerto Ricans had higher overweight/obesity than Cubans [7]; Japanese, Filipino, and other Asian subgroups had higher overweight/obesity than Chinese [8], [9]. In U.S. adolescents, patterns are somewhat similar. In one study, obesity was more common in Puerto Rican than in Mexican and Cuban adolescents [10]; in another, Mexican, Puerto Rican, and Cuban adolescents had similar obesity prevalence which was higher than in Central/South Americans [11]. In Asians, obesity was higher in Asians of other ancestral background compared to Chinese and Filipino adolescents [11].

While there are clear ethnic subgroup disparities in obesity among Hispanics and Asians in the U.S., no studies to our knowledge have examined longitudinal changes in body mass index (BMI) by ethnic subgroup to understand how and when these disparities emerge, especially during the critical transition between adolescence and adulthood. Using longitudinal data from the National Longitudinal Study of Adolescent Health (Add Health), we examined whether Hispanic and Asian adolescent subgroups shared the same BMI trajectory as white adolescents as they aged into adulthood. This allowed us to evaluate whether there are sensitive periods in the life-course for the emergence of BMI disparities and if these sensitive periods differ across Hispanic and Asian subgroups. Consistent with past cross-sectional findings, we hypothesized that among Hispanics, Mexican and Puerto Rican respondents would exhibit faster increases in BMI from adolescence to adulthood relative to whites, and among Asians, Chinese respondents would exhibit slower BMI increases. We also investigated the contribution of social and behavioral risk factors in explaining observed race/ethnic differences in adolescent BMI and in BMI change over time. Hispanics and Asians are projected to constitute an increasing share of the U.S. population [12], [13]. Therefore, a more nuanced understanding of the health patterns that emerge will be important for the design of more effective interventions.

## Materials and Methods

### Ethics

Data for the Add Health cohort were collected under protocols approved by the Institutional Review Board at the University of North Carolina at Chapel Hill. Written parental/guardian consent and adolescent assent were obtained before the Wave I interview. At Wave IV, written consent was obtained from all respondents.

### Data

The Add Health cohort is a nationally representative school-based study of adolescents (n = 20,745; age 11–20 years), in grades 7 to 12 in 1994–95 (wave I) who were followed into adulthood. Information on how to obtain the Add Health data files is available on the Add Health website (http://www.cpc.unc.edu/addhealth). The study used a multistage, stratified, school-based, clustered sampling design and included interviews with 85% of the respondents’ parents. Additional subsamples were also drawn to provide meaningful data on individuals of Chinese, Cuban, and Puerto Rican origin. Details regarding the survey design and sampling frame have been previously described [14]. Of the 20,745 adolescents surveyed in wave I, 14,738 participants in grades 7–11 were re-interviewed at wave II in 1996 (age: 12–21 years). At wave III in 2001–02 (age: 18 to 27 years; n = 15,197) and wave IV in 2008–09 (age: 24–33 years; n = 15,701), all wave I respondents were eligible for follow-up regardless of wave II participation. Non-response analysis indicates no significant bias to Add Health estimates from attrition across waves [15].

The analysis sample included white, Hispanic, and Asian respondents interviewed in wave II with longitudinal, post-stratification sample weights (n = 7308). Exclusions included respondents with missing measured height and weight at wave II (height and weight were not measured at wave I), and pregnant females, yielding a final analytic sample of 6936 adolescents from the following race/ethnic groups: 5061 whites, 745 Mexicans, 204 Puerto Ricans, 198 Cubans, 115 Central/South Americans, 93 Other Hispanics (Dominicans and Hispanics of mixed ancestry), 175 Chinese, 252 Filipinos, 93 Other Asians (combination of Koreans, Japanese, Vietnamese, and Asian Indians). Respondents contributed anywhere from 1 to 3 height and weight observations (wave II – wave IV; mean = 2.9 observations across 6936 individuals) for longitudinal analyses (mean follow-up of 12 years).

### Measures

#### Outcome

Height (m) and weight (kg) were measured at waves II through IV during in-home surveys using standardized procedures. Before computing BMI (weight (kg)/height (m)^2^), we excluded implausible height (n = 44) and weight (n = 17) values. In the case where height change declined by >4 inch inches (n = 158), we substituted stable adult height across visits using mean values from exams where males were >17 years and females were >15 years, or set values to missing if no such height measures were available at these ages. We utilized repeated, continuous measures for the dependent variable, BMI, in longitudinal models. Even though BMI z-scores have been identified as the optimal measure of body mass at a single time point for children, raw BMI scores are recommended to evaluate change in body mass during childhood and adolescence in longitudinal studies. [16]–[18] Briefly, the CDC/NCHS reference curves are based on repeated cross-sections and thus do not adequately capture individual growth patterns. Furthermore, given the heterogeneity in our cohort and the fact that the CDC/NCHS reference curves do not adequately capture this ethnic diversity in Hispanics and Asians – the primary groups of interest in our study, BMI Z-scores are not ideal.

#### Independent variables

Data on ethnicity and country of origin/ancestry were obtained by respondent and parental self-report at wave I. A single race/ethnicity variable was created and used to compare BMI patterns across Hispanic and Asian subgroups relative to the non-Hispanic white referent group. Immigrant generation was based on adolescents’ and parents’ place of birth. Generation one included children not born on the mainland U.S., Alaska or Hawaii. Although Puerto Ricans are U.S. citizens by birth, we classified Puerto Rican-born respondents as first generation as per the established acculturation-based approach [11], [19]. Generation two or greater included U.S.-born children with either foreign-born or U.S.-born parents.

Markers of childhood socioeconomic status (SES) included parental education and welfare receipt prior to age 18 years. Parents’ education was measured as the higher of either the mother’s or father’s education: less than high school, high school/GED, some college, and college degree or more. Welfare receipt was constructed from data on the family’s receipt of public assistance/welfare from waves I and II, in combination with a retrospective report at wave III.

#### Lifestyle behaviors

We also explored whether behaviors known to influence adolescent BMI and BMI change could explain observed ethnic differences. Current smoking (yes/no), screen time (hours of TV, video, and computer game use per week), and physical activity (weekly bouts of moderate to vigorous physical activity) were ascertained at all waves. These measures were based on standard, interview-administered questionnaires validated in other epidemiologic studies [20]. Using responses from the waves I and II In-Home Questionnaires, we also ascertained whether respondents regularly skipped breakfast (yes/no).

Other controls: respondents’ age at each exam and sex (male/female).

### Statistical Analysis

Analyses were performed using Stata software, version 12.1 (Stata Corp, College Station, Texas). Descriptive analyses used post-stratification sample weights to reflect national population estimates. Adjusted Wald tests compared means and design-based F-tests compared the distribution of categorical variables across ethnic groups. All analyses used multiple stages of cluster sampling to adjust for survey design effects.

We used mixed linear spline models with the unstructured covariance specification to estimate Hispanic and Asian subgroup differences in mean baseline BMI and in BMI change, relative to non-Hispanic whites, as adolescents aged into adulthood. Mixed models are well suited for studies of individual changes over time, using repeated measures of an outcome to estimate a growth trajectory defined by an intercept (baseline) and a slope (rate of change). These models allow for missing outcome data, so that all participants with at least one BMI measure were included in the analyses. Age (in years) represented the measure of time.

Smoothing splines were used to characterize the non-linear relationship between age and BMI. Various numbers and positions of knots were compared to find the best fit for the data. The optimal model had one knot placed at age 20 years, based on a comparison of the Akaike Information Criterion (AIC) across the different models tested. Age was centered at the earliest observed age, so that the model intercept represented mean BMI at age 12. The slope represents the change in BMI per increase in year of age. Random effects at the individual level were included for the intercept and linear age term, allowing both intercept and linear slope to vary between individuals.

The base model included the race/ethnicity variable and interactions between race/ethnicity and the linear age and age spline terms, adjusting for sex and study site region. The coefficient for the race/ethnicity term tested whether, at age 12, mean BMI differed across the five Hispanic and three Asian subgroups compared to non-Hispanic whites. The interactions tested whether the BMI trajectories after age 12 (linear age term), and after age 20 (age spline term), differed by race/ethnicity. We subsequently introduced predictors for immigrant generation, SES, and behaviors in sequential models to investigate whether these social and behavioral factors explained observed ethnic differences in BMI. We assessed potential confounding by evaluating whether their inclusion changed model estimates. All covariates, except for time-varying variables, were also interacted with the age terms. Continuous variables (screen time and physical activity) were centered on their grand means to facilitate model interpretation. Interaction terms between sex and race/ethnicity at baseline and with the age terms were also tested to investigate whether race/ethnic patterns differed for males and females. In sensitivity analyses, we restricted the sample to respondents <16 years of age at baseline. Although some ethnic-specific estimates were no longer significant, the magnitude and direction of the associations were consistent with the results we report for the overall sample (results not shown).

## Results

### Descriptive Statistics

#### Hispanics

At the youngest ages (12–14 years), mean BMI was similar across subgroups compared to whites (Table 1). However BMI increased with age to a greater extent among Mexican and Puerto Rican origin respondents. Central/South Americans had the highest proportion of first generation immigrants whereas Puerto Rican adolescents were primarily U.S.-born. All Hispanics were disproportionately concentrated in lower SES groups than whites with variability by subgroup (e.g. 53% of Mexican adolescents had parents with less than high school education; Central/South American adolescents had the highest proportion of parents with at least a college degree (22%)). Ethnic differences were apparent for some health behaviors. For example, smoking rates were lower for all Hispanic subgroups compared to whites in adolescence and in adulthood. Puerto Ricans were the exception as smoking rates were comparable to whites in adolescence, and exceeded rates among whites in adulthood.

**Table 1:** Sample Characteristics: Means (SE) and Weighted Frequencies by Race/ethnic Subgroups, National Longitudinal Study of Adolescent Health.^a.^

		HISPANICS					ASIANS		
	Non-Hispanic Whites (n = 5187)	Mexicans (n = 762)	Puerto Ricans (n = 212)	Cubans (n = 202)	Central/South Americans (n = 118)	Other Hispanics (n = 96)	Chinese (n = 178)	Filipinos (n = 259)	Other Asians (n = 93)
**Mean BMI (kg/m^2^) by age, years**									
12–14	22.00 (.23)	22.05 (.50)	21.94 (.42)	23.35 (.90)	21.89 (.63)	20.85 (0.98)	19.78 (.42)****	21.03 (.96)	18.82 (.49)****
15–19	23.41 (.13)	24.08 (.25)**	25.21 (.52)***	23.51 (.58)	23.72 (.48)	23.18 (.60)	21.45 (.45)***	22.66 (.42)*	20.91 (0.34)****
20–24	25.94 (.16)	27.29 (.33)***	27.18 (.53)**	27.84 (1.34)	26.45(.52)	25.03 (.61)	23.38 (.49)****	25.46 (.50)	23.53 (.50)****
25–33	28.34 (.19)	29.84 (.41)***	30.41 (.54)***	28.73 (.39)	28.55 (.54)	27.21 (.80)	25.08 (.46)****	27.23 (.59)*	24.24 (0.42)****
**Mean BMI change, kg/m^2^**	6.35 (.23)	7.78 (0.45)***	8.47 (0.60)***	5.38 (0.89)	6.66 (0.71)	6.36 (1.07)	5.30 (0.53)*	6.19 (0.76)	5.42 (0.61)
**%Female**	48.35	45.08	43.09	55.05	51.07	51.75	43.73	38.48	45.15
**Immigrant generation, %** ****									
1^st^	0.49	16.84	6.02	25.66	54.18	23.91	30.51	44.18	39.89
2nd or more	99.51	83.16	93.98	74.34	45.82	76.09	69.49	55.82	60.11
**Parental Education, %** ****									
Less than HS	8.82	52.79	25.81	32.78	29.06	25.91	17.14	4.35	13.73
HS	32.88	22.18	31.62	31.74	24.09	27.07	22.34	15.57	26.36
Some college	30.34	16.27	27.79	21.69	24.47	28.91	13.11	22.92	19.63
College or more	27.96	8.77	14.78	13.79	22.37	18.11	47.41	57.16	40.28
**Welfare receipt before age 18, %** ****	24.61	39.70	45.26	35.05	19.77	33.70	15.10	21.14	18.85
**Skip breakfast, %**	11.18	11.46	11.68	8.87	10.19	13.49	12.94	10.36	10.82
**Mean screen time** b **(hours/wk)**	15.53 (0.28)	16.88 (0.68)*	16.66 (1.05)	14.70 (0.55)	16.66 (0.94)	14.80 (0.96)	13.50 (1.23)	17.81 (0.99)**	13.56 (1.02)*
**Mean physical activity** c **(bouts/wk)**	4.22 (0.05)	4.06 (0.13)	4.41 (0.29)	3.83 (0.21)*	3.75 (0.27)*	4.19 (0.27)	4.39 (0.30)	4.35 (0.22)	4.09 (0.30)
**Current smoking** d **, %**									
Adolescence****	36.94	27.02	37.52	9.11	23.56	27.42	13.31	27.12	16.52
Adulthood****	41.59	26.60	47.97	20.29	27.17	26.96	22.24	29.06	25.80

Abbreviations: SE, standard error; BMI, body mass index; HS, high school.

**** <0.0001;

*** <0.01;

** <0.05;

* <0.1, P-value comparing Hispanic and Asian subgroups to whites.

a Weighted for national representation, standard errors corrected for survey design effects of multiple stage cluster sampling.

b Screen time = hours of TV, video, and computer game use per week; estimates averaged across waves (wave II–IV).

c Physical activity = weekly bouts of moderate to vigorous physical activity; estimates averaged across waves (wave II–IV).

d Current smoking: any cigarette smoking in previous 30 days; adolescence: age <18; adulthood: age > = 18.

#### Asians

Chinese and Other Asians had significantly lower mean BMI than whites at 12–14 years of age, which remained lower with increasing age (Table 1). Although adolescent BMI among Filipinos was slightly lower than whites, this difference was not statistically significant. Nevertheless, there was some evidence that mean BMI increased into adulthood at a slower rate for Filipinos than for whites. Chinese individuals were more likely to be U.S.-born than Filipinos or Other Asians. All subgroups had a higher proportion of parents with a college degree than whites. Notable differences in health behaviors included a lower proportion of smokers among all Asian subgroups compared to whites.

### Multi-variable Analyses

**Figure 1:**
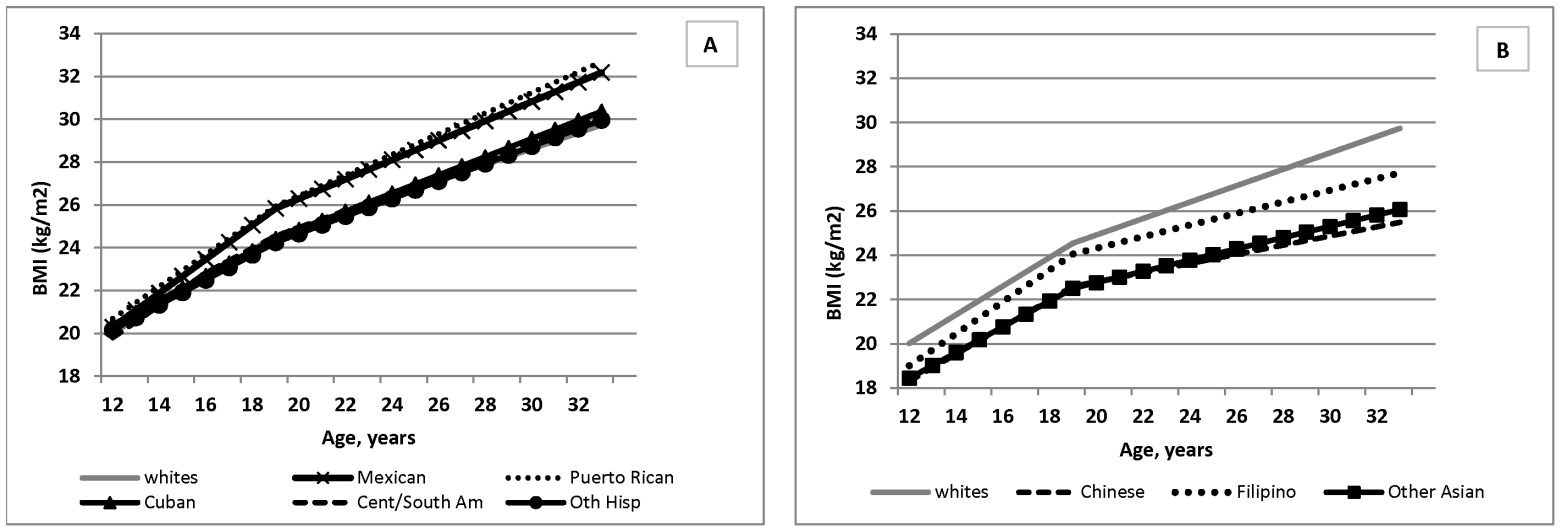
Mean BMI trajectories by Hispanic and Asian subgroups compared to whites, National Longitudinal Study of Adolescent Health. A) Hispanic subgroups vs. whites, B) Asian subgroups vs. whites. Predicted BMIs are derived from coefficients from Model 1, Table 2 (adjusted for sex and study region).

In age, sex, and region-adjusted models, there were no significant differences in mean BMI at age 12 for any of the Hispanic subgroups compared to whites (Model 1, Table 2 and Figure 1A). However the significant, positive coefficients for the BMI slopes for Mexicans (MX) and Puerto Ricans (PR) relative to whites indicated a faster rise in BMI from age 12 to <20 years (hereafter referred to as ‘adolescence’) (MX: β = .15 kg/m^2^, SE = .03; PR: β = .10 kg/m^2^, SE  = .06) and from age 20 years and on (hereafter referred to as ‘adulthood’) (MX: β = 0.08 kg/m^2^, SE = .02; PR: β = .11 kg/m^2^, SE  = .04). Although BMI tended to increase more slowly for Cubans and faster for Central/South Americans in adolescence, there was little difference in the rate of BMI growth after the age of 20 years compared to whites.

**Table 2:** Adjusted Mean Difference in Adolescent BMI and BMI Change from Adolescence to Adulthood by Race/ethnic Subgroups.

	Model 1			Model 2			Model 3			Model 4		
		Growth rate			Growth rate			Growth rate			Growth rate	
	Baseline(Age = 12 years)	Age(<20 years)	Age(> = 20 years)	Baseline(Age = 12 years)	Age(<20 years)	Age(> = 20 years)	Baseline(Age = 12 years)	Age(<20 years)	Age(> = 20 years)	Baseline(Age = 12 years)	Age(<20 years)	Age(> = 20 years)
Intercept	20.02 (.22)	.65 (.02)****	.37 (.01)****	20.01 (.22)	.65 (.02)****	.37 (.01)****	20.31 (.33)	.64 (.05)****	.41 (.03)****	20.69 (.35)	.59 (.05)****	.42 (.03)****
**Race/ethnicity (ref = non-Hispanic whites)**												
HISPANICS												
Mexican	.27 (.29)	.15 (.03)****	.08 (.02)****	.35 (.26)	.16 (.03)****	.09 (.02)****	.02 (.30)	.16 (.04)****	.05 (.02)**	−0.08 (.29)	.16 (.04)****	.06 (.02)**
Puerto Rican	.67 (.47)	.10 (.06)*	.11 (.04)***	.69 (.47)	.11 (.05)**	.11 (.04)***	.49 (.47)	.11 (.06)**	.09 (.04)**	.35 (.47)	.12 (.06)**	.10 (.04)**
Cuban	.39 (.26)	−.07 (.02)***	.05 (.04)	.60 (.36)*	−.04 (.02)	.06 (.04)	.46 (.34)	−.04 (.03)	.05 (.04)	.27 (.32)	−.02 (.02)	.05 (.04)
Central/South American	−.22 (.61)	.11 (.10)	.03 (.06)	.09 (.58)	.16 (.09)*	.04 (.05)	.05 (.57)	.16 (.09)*	.03 (.05)	−.13 (.56)	.18 (.09)*	.03 (.05)
Other Hispanics	.14 (.59)	−.06 (.09)	.04 (.05)	.22 (.60)	−.04 (.09)	.04 (.05)	.03 (.60)	−.04 (.09)	.02 (.05)	−.03 (.64)	−.05 (.10)	.02 (.05)
ASIANS												
Chinese	−1.71 (.40)****	−.04 (.05)	−.16 (.04)****	−1.54 (.42)****	−.01 (.04)	−.16 (.04)****	−1.43 (.41)***	−.01 (.04)	−.15 (.03)****	−1.61 (.43)****	−.01 (.05)	−.15 (.03)****
Filipino	−1.01 (.39)**	.07 (.06)	−.11 (.03)***	−.70 (.41)*	.12 (.08)	−.10 (.03)***	−.58 (.36)	.12 (.07)	−.07 (.02)***	−.76 (.34)**	.13 (.06)**	−.08 (.02)***
Other Asian	−1.58 (.75)**	−.07 (.12)	−.12 (.03)****	−1.39 (.76)*	−.04 (.13)	−.11 (.04)***	−1.30 (.77)**	−.04 (.13)	−.10 (.04)***	−1.42 (.75)*	−.03 (.13)	−.08 (.04)**
**Female**	−.13 (.13)	−.05 (.02)**	−.02 (.01)*	−.13 (.13)	−.05 (.02)**	−.02 (.01)*	−.07 (.14)	−.05 (.02)**	−.02 (.01)*	−.11 (.15)	−.05 (.02)**	−.03 (.01)**
**Immigrant generation (ref = 2^nd^ or more)**												
1^st^				−.52 (.41)*	−.10 (.05)**	−.02 (.03)	−.59 (.40)**	−.10 (.05)**	−.03 (.03)	−.48 (.37)	−.11 (.05)**	−.03 (.03)
**Parental education (ref = less than high school)**												
High school							−.19 (.28)	.02 (.05)	−.02 (.03)	−.16 (.27)	.02 (.05)	−.02 (.03)
Some college							−.35 (.29)	−.01 (.05)	−.04 (.03)*	−.29 (.29)	−.01 (.05)	−.05 (.03)*
College or more							−.74 (.29)**	.01 (.04)	−.10 (.03)****	−.64 (.29)**	.01 (.04)	−.10 (.03)****
**Welfare receipt before 18 years of age (ref = no)**							.61 (.19)***	.0004 (.03)	.03 (.02)	.56 (.19)***	−0.002 (.03)	.03 (.02)
**Skip breakfast (ref = no)**										.82 (.30)***	.11 (.05)**	.05 (.02)**
*Time-varying behaviors*												
**Screen time**										.01 (.002)****		
**Moderate/vigorous physical activity**										−.05 (.008)****		
**Smoking status (ref = no)**										−.34 (.06)****		

Abbreviations: BMI, body mass index; SE, standard error.

**** <0.0001;

*** <0.01;

** <0.05;

* <0.1 (P-value).

Model 1 adds age spline terms, sex, and study region; Model 2 adds immigrant generation; Model 3 adds socioeconomic factors: parental education and household welfare receipt prior to 18 years of age; Model 4 adds health behaviors: time-varying variables for moderate/vigorous physical activity, screen time, and current smoking status; and skipping breakfast (wave II).

Among Asians, mean BMI at baseline (age 12 years) was significantly lower than whites for all Asian subgroups: Chinese (β = −1.71 kg/m^2^, SE = .40), Filipinos (β = −1.01 kg/m^2^, SE = .39), and Other Asians (β = −1.58 kg/m^2^, SE = .75) (Model 1, Table 2 and Figure 1B). Although results indicated no significant differences in the rate of BMI change in adolescence compared to whites for all Asians, the magnitude of the estimate for Filipinos suggested a slightly faster rise for this subgroup (β = 0.07 kg/m^2^, SE = .06). After age 20 years, BMI increased to a significantly slower extent for all Asians compared to whites.

In sequential models, we added immigrant generation (Model 2), parental SES (Model 3), and health behaviors (Model 4). After adjusting for the higher proportion of immigrants among many of the Hispanic subgroups (Model 2, Table 2), mean difference estimates in baseline BMI increased for Cubans and Central/South Americans. In other words, if not for the health ‘protective’ influence of immigrant status, compared to whites, Cubans would have higher mean BMI at age 12 years, and Central/South Americans would have more similar mean BMI values. Central/South Americans also exhibited faster increases in BMI in adolescence after adjusting for immigrant status (β = 0.16 kg/m^2^, SE = .09).

In Asians, immigrant status partially attenuated mean BMI differences at age 12 in all subgroups, but to a greater extent among Filipinos. While not statistically significant, the larger BMI slope estimate also pointed to an increase in the magnitude of the BMI change during adolescence for Filipinos relative to whites. After adjusting for the high proportion of low SES across Hispanic groups, mean differences in BMI at age 12 decreased appreciably for all subgroups, but there was little impact on longitudinal estimates (Model 3, Table 2). In all Asians, there was additional attenuation of BMI differences at baseline with the addition of parental SES, though these estimates remained statistically significant for all subgroups except Filipinos.

Skipping breakfast, higher screen time, lower physical activity, and not currently smoking were all highly associated with higher BMI (Model 4, Table 2). Among Hispanics, although baseline ethnic differences in BMI were even further attenuated, there was little change to longitudinal estimates. In other words, the faster BMI increases in adolescence and adulthood for Mexicans and Puerto Ricans, and in adolescence for Central/South Americans, remained unexplained after inclusion of social and behavioral covariates. For Asians, rather than attenuating estimates, accounting for lifestyle behaviors further magnified BMI differences at age 12 years among Chinese, Filipinos, and Other Asians compared to whites. These behaviors also did not explain the faster BMI increase in adolescence among Filipinos, or the slower BMI increases in adulthood exhibited by all Asian subgroups relative to whites.

**Figure 2:**
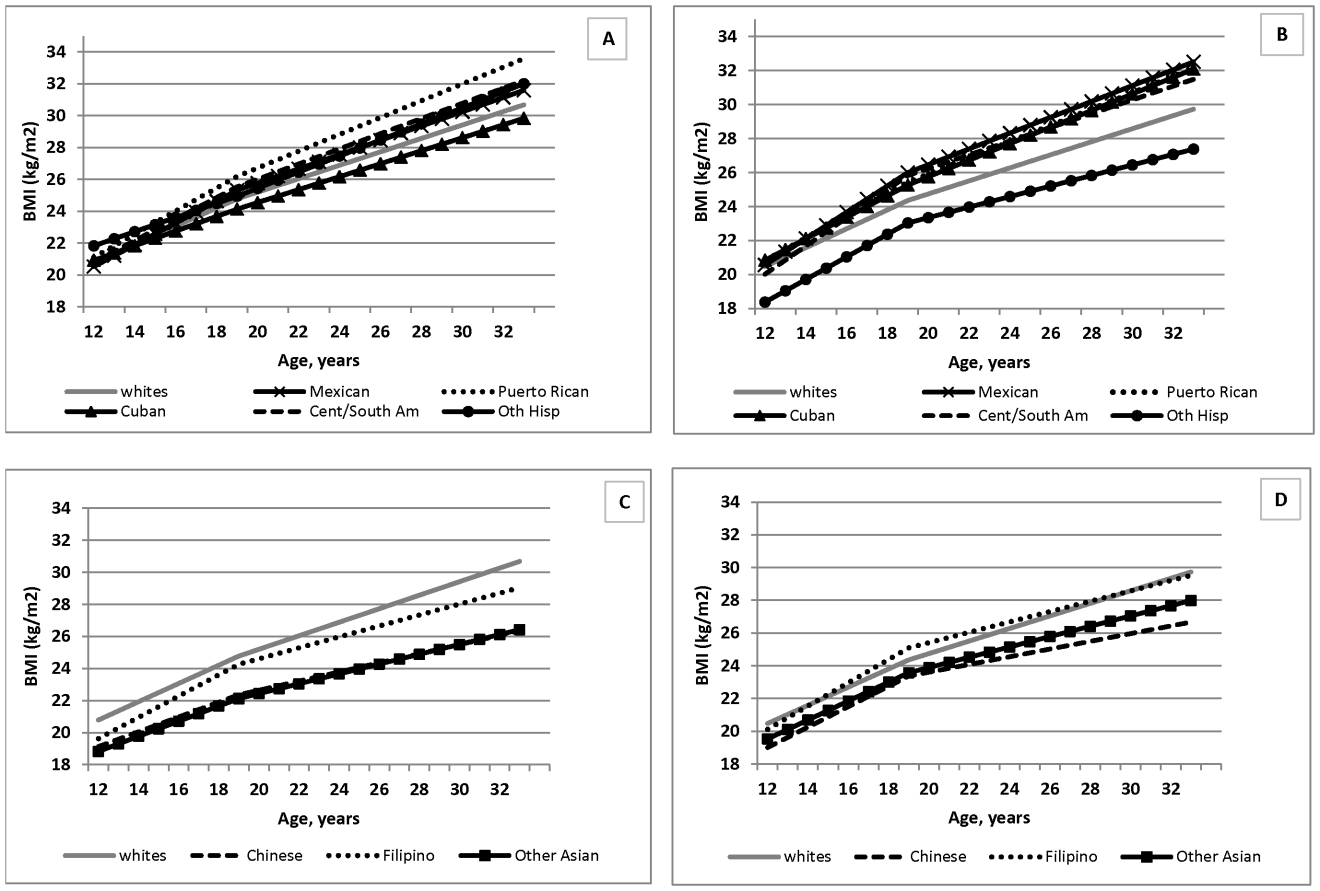
Adjusted BMI trajectories by Hispanic and Asian subgroups compared to whites, females and males, National Longitudinal Study of Adolescent Health. A) Hispanic subgroups vs. whites - Females, B) Hispanic subgroups vs. whites - Males, C) Asian subgroups vs. whites – Females, D) Asian subgroups vs. whites - Males. BMI = body mass index. Predicted BMIs are based on adjusted model from Model 4, Table 2, stratified by sex.

Some race/ethnic patterns in baseline (age 12 years) BMI and BMI change differed by sex in the fully adjusted model (Model 4; P-interaction <0.0001). To facilitate interpretation, predicted values using coefficients from this fully-adjusted model stratified by sex were plotted in separate figures for Hispanic (Figures 2A, 2B) and Asian (Figures 2C, 2D) females and males compared to whites. Among Hispanic females, there were no race/ethnic differences in adolescent BMI after covariate adjustment as in the overall sample (Figure 2A), but among males, Other Hispanics had significantly lower BMI at age 12 years than whites (Figure 2B). During the transition to adulthood, although Cuban females had similar rates of BMI increase as white females, consistent with findings from the main effects model, Cuban males exhibited significantly larger increases than white males during both adolescence and adulthood. By the time respondents reached adulthood, all Hispanic females, except Cubans, had higher BMI than white females, and all Hispanics males, except Other Hispanics, had higher BMI than white males. Among Asians, the lower adolescent BMI and slower BMI increases in Chinese relative to whites occurred to a significantly greater extent in females than males (Figures 2C, 2D).

## Discussion

We investigated whether BMI trajectories differed across subgroups of Hispanics and Asians relative to white adolescents transitioning to adulthood and evaluated what social and behavioral factors might underlie these disparities. Among Hispanics, Mexican and Puerto Rican-origin individuals exhibited faster increases in BMI both in adolescence and in adulthood and these patterns were not attributable to the social and behavioral factors we measured. There was also evidence of emerging disparities among Cuban males, and among Central/South Americans relative to whites In Asians, the pattern previously reported in the literature whereby Asians overall had lower BMI than whites was observed here for all three subgroups – Chinese, Filipino, and Other Asians - in age and sex-adjusted models. All Asians also experienced slower BMI increases in adulthood relative to whites. However in adolescence, the BMI of Filipinos appeared to converge to levels found in whites. This was especially evident after accounting for immigrant status and SES which also attenuated BMI differences between Filipinos and whites at age 12 years. BMI differentials between Chinese and whites and Other Asians and whites, however, remained unexplained.

Previous studies in young children have documented higher BMI and faster BMI gains among Hispanics relative to whites and Blacks. In an analysis of data from the Early Childhood Longitudinal Study, the largest BMI gains during the elementary school years occurred between 1^st^ and 3^rd^ grade; and across race/ethnic groups, Hispanic boys and girls had the most excess gains relative to whites [21]. Other work also showed that among children aged 6–11 years, Mexican-American boys and girls had higher obesity prevalence than whites, and among boys, prevalence was also higher in Mexican-Americans than Blacks [22].

Much of this research has examined Hispanics as a single group, or has reported findings that pertain only to Mexican-Americans. Studies that have investigated BMI differences by subgroup have been entirely cross-sectional [10], [11], and thus are unable to examine when disparities emerge and how they pattern into adulthood within the same individual. Our findings suggest that, especially for Hispanics, BMI disparities relative to whites tend to be concentrated among Mexican and Puerto Rican origin individuals, regardless of sex, and these disparities considerably worsen as they get older. The adverse patterns we report for Cuban males throughout adolescence and adulthood and among Central/South Americans in adolescence also points to the emergence of disparities for other Hispanic subgroups as well.

While BMI at age 12 did not differ significantly from whites, the faster BMI increases in adolescence among Mexicans and Puerto Ricans suggests that factors during and prior to this life stage may contribute to adverse body mass patterning for the two largest U.S. Hispanic subgroups [12]. However despite adjustment for several social and behavioral covariates, we could not account for the steeper BMI trajectories we observed. Conversely, we also could not explain why Chinese and Other Asian adolescents in particular had a lower adolescent BMI and a slower trajectory of increase. Change in body mass, at a proximal level, is a function of health behaviors linked to energy intake and expenditure. Although we had time-varying information available on key determinants of adolescent weight gain, such as physical activity, screen time, and smoking, one major limitation of this work was the lack of 24-hour dietary recall information and/or longitudinal dietary data which may have better accounted for race/ethnic differences in BMI trajectories.

We also cannot rule out the role of genetic factors in driving susceptibility to increase in body mass among some Hispanics, and the differential susceptibility among Chinese and Other Asians relative to whites. Contemporary research is in the process of disentangling the role of genetic influences, particularly as they relate to the propensity for obesity and weight gain [23]. However the population heterogeneity of Hispanics and Asians poses a challenge for genetic health studies of nationalities or ethnic groups since within-group genetic heterogeneity can arguably be greater than across race/ethnic groups [24]. As a result, rather than explain race/ethnic disparities, genetic markers may instead be useful for investigating within-race/ethnic group differences to further identify at-risk subgroups.

Beyond biological factors, adolescent body mass and its change over time are also strongly influenced by social, cultural, and other environmental factors that we did not account for [25]. For example, there is research to suggest that there may be cultural factors linked to perception of ideal body weight [26], [27], and differential access to physical environmental and health-promoting resources [28]–[30], which may also contribute to ethnic differences in BMI trajectories from adolescence into adulthood. Future research should consider the role that such factors play in race/ethnic subgroup patterning of BMI.

There were some additional limitations. Although Add Health over-sampled select ethnic groups, we nevertheless had small sample sizes for some categories which may have limited statistical power to test associations. Along these lines, grouping ‘Other Asians’ and ‘Other Hispanics’ also likely masked important associations for distinct ethnicities such as Japanese, Indian-Americans, Vietnamese, etc., and for Hispanic ethnicities such as Dominicans. Just as there is heterogeneity by ethnicity among Hispanics and Asians, there is also ethnic heterogeneity among whites that we did not account for.

There were also other potential explanatory factors that we did not assess. Early life circumstances associated with fetal under-nutrition, low birthweight, and infant formula feeding have been associated with rapid growth in infants and young children, predisposing them to adult obesity and its associated risks [31], especially when exposed to environments that promote positive energy balance. Future work should also assess the role of potential early life determinants in explaining race/ethnic disparities in BMI.

Overlooking within-group heterogeneity in the BMI trajectories of Hispanics and Asians in the transition from adolescence to adulthood masks important differences regarding how and when disparities emerge. We noted the development of BMI disparities for Mexicans and Puerto Ricans relative to whites that appeared to emerge in adolescence and widened into adulthood, and of a disparity that extended to other subgroups like Cuban males and to a lesser extent, among Central/South Americans. Obesity has consequences for diabetes and other metabolic abnormalities, and Hispanics have been shown to have among the highest risk of developing such complications [32]. As a result, our findings that most Hispanic subgroups were at risk of more rapid gains in BMI in the transition from adolescence to adulthood when the antecedents for chronic disease develop, will have implications for future chronic disease burden in the U.S., and for exacerbating Hispanic-white disparities in adulthood. For Asians, ‘protection’ against higher BMI in adolescence among Filipinos was largely attributable to their socio-demographic profile. However social and behavioral factors did not account for Chinese-white and Other Asian-white differentials. Despite their lower BMI, Chinese-origin individuals in particular have been shown to be at increased risk for diabetes at lower adiposity thresholds than other race/ethnic groups [33], [34]. Thus, the lower baseline BMI and slower BMI increase we report for Chinese does not necessarily reflect a lower risk of developing cardiovascular disease. Future research should seek to understand what other factors may underlie existing race/ethnic disparities in BMI. Identification of these factors will allow for improved targeting of obesity prevention efforts in ethnically-diverse youth.
